# mRNA-Enhanced Cell Therapy and Cardiovascular Regeneration

**DOI:** 10.3390/cells10010187

**Published:** 2021-01-19

**Authors:** Palas K. Chanda, Roman Sukhovershin, John P. Cooke

**Affiliations:** RNA Therapeutics Program, Center for Cardiovascular Regeneration, Department of Cardiovascular Sciences, Houston Methodist Research Institute, 6670 Bertner Ave., Houston, TX 77030, USA; pkchanda@houstonmethodist.org (P.K.C.); rsukhovershin@houstonmethodist.org (R.S.)

**Keywords:** RNA therapeutics, cell therapy, cardiovascular regeneration, inflammatory signaling, nuclear reprogramming, iPSCs, transdifferentiation, cardiovascular ageing

## Abstract

mRNA has emerged as an important biomolecule in the global call for the development of therapies during the COVID-19 pandemic. Synthetic in vitro-transcribed (IVT) mRNA can be engineered to mimic naturally occurring mRNA and can be used as a tool to target “undruggable” diseases. Recent advancement in the field of RNA therapeutics have addressed the challenges inherent to this drug molecule and this approach is now being applied to several therapeutic modalities, from cancer immunotherapy to vaccine development. In this review, we discussed the use of mRNA for stem cell generation or enhancement for the purpose of cardiovascular regeneration.

## 1. Introduction

Advances in the biopharmaceutical industry were accelerated in the global race toward therapies for the COVID-19 pandemic [1]. Most notably, messenger ribonucleic acid (mRNA) vaccines galvanized the field, with lightspeed generation of new therapeutic molecules. For example, within 42 days of the publication of the SARS-CoV-2 sequence by Chinese scientists in January 2020 [2], Moderna sent its RNA vaccine candidate to the National Institute of Allergy and Infectious Disease for preclinical testing. By April 2020, Moderna launched its first clinical trial. Less than 8 months later, Moderna will be seeking Emergency Use Authorization for its vaccine, after phase III trials revealed 95% efficacy and excellent safety. Out of the 236 COVID-19 vaccines being developed, 29 of them are mRNA-based [1] and the first two (BNT162 from Pfizer and MRNA1273 from Moderna) of all the vaccines to complete the phase III clinical trial belong to this category [3,4]. These vaccines will be the first mRNA therapeutics to reach the market. The speed by which mRNA vaccines were developed, and their high degree of efficacy and safety, has brought attention to the great promise of mRNA therapeutics.

Whereas the majority of drugs approved by U.S. Food and Drug Administration (FDA) are small molecules, such drugs have limitations in the range of diseases that are “druggable” [5,6]. In contrast, mRNA has nearly limitless range, as this biological software can be rapidly modified to encode any therapeutic protein or antigen of interest. Furthermore, with advances in delivery methods, pharmacokinetic and pharmacodynamic properties, enhanced efficacy and stability and reduced immunogenicity and production costs [7,8], mRNA therapeutics have an almost limitless potential.

mRNA therapeutics offers several advantages over the contemporary small molecule, protein or DNA-based therapies. For example, it is difficult to generate small molecules that will allosterically enhance the activity of a deficient enzyme. It may also be difficult to generate a properly folded and post-translationally modified recombinant protein for the same deficit. By contrast, mRNA encoding the wild-type enzyme is easily generated, and when delivered to the appropriate cell type, can replace the deficient enzyme. Compared to DNA delivered gene vectors, RNA is biologically active in both dividing and non-dividing cells [9] and does not need to enter the cell nucleus to generate its therapeutic effect. Furthermore, with standard mRNA, there is no risk of altering the host genome. Synthetic in vitro-transcribed (IVT) mRNA is designed to mimic naturally occurring mRNA [10], i.e., a single-stranded open reading frame flanked by untranslated regions, a 5′ cap for translation, and a 3′ poly(A) tail for stability [11,12]. Modified nucleosides (e.g., 5-methylcytosine and pseudouridine) are included to reduce the cellular toxicity associated with immunogenicity of exogenous mRNA [13]. Furthermore, the mRNA sequence can be modified to include synonymous codons that are optimal for a specific cell type (reflecting tRNA abundance), or a “translational ramp” (specific initial amino acid sequence that enhances translation) [14,15].

An mRNA-based approach lends itself to a number of therapeutic modalities, including (a) replacement therapy to compensate for a defective gene/protein, or to supply therapeutic proteins [16]; (b) vaccination, where mRNA encoding specific antigen(s) is administered to trigger protective immunity [16]; (c) cell therapy, which involves transfection of mRNA into the cells ex vivo to therapeutically enhance cell survival, proliferation and/or function [17]; (d) generation of new monoclonal antibodies using mRNA [18]; (e) gene editing, where mRNA is used to express an enzyme that edits and corrects the disease-causing defective gene [19].

Advances in mRNA design, production and delivery has sparked the exploration of mRNA therapy in different fields, such as immunotherapy against cancer and infectious diseases [20], production of growth factors, generation of engineered mesenchymal stem cells (MSCs) and regenerative medicine. In this review, we will focus on the use of mRNA for stem cell generation or enhancement, for the purpose of cardiovascular regeneration.

## 2. Employing mRNA to Generate iPSCs for Stem Cell Therapy

In 2006, Yamanaka and colleagues [21] demonstrated that terminally differentiated adult somatic cells can be reprogrammed to generate induced pluripotent stem cells (iPSCs) by ectopic expression of a specific set of transcription factors, *Pou5f1*, *Sox2*, *Klf4* and c-*Myc* (OSKM) using retroviral vectors. These iPSCs are highly similar to embryonic stem cells (ESC) in terms of self-renewal and the ability to be differentiated to all three germ layers. Thus, human iPSCs can be used as an alternative for human ESCs, thereby avoiding potential ethical issues. This seminal discovery was transformative for the field of regenerative medicine. Patient-specific iPSCs generated from somatic cells can be differentiated to understand the pathobiology of genetic diseases. Furthermore, differentiated derivatives of iPSCs could serve as therapeutic cells. For example, iPSCs generated from a patient with a genetic disease (such as cardiomyopathy due to muscular dystrophy) could undergo ex vivo genome editing [22]. These iPSCs could be differentiated into therapeutic cells which could be transplanted into the patient with minimum risk of genetic incompatibility or immune rejection.

However, viral vectors (retroviral, lentiviral or adenoviral vectors) used to generate iPSCs confer risk of genomic integration and limit the clinical application of such iPSCs [23]. Consequently, several integration-free approaches have been developed, including the Sendai virus [24], cell permeant recombinant proteins [25], non-integrating plasmids or episomal DNA [26,27]. Although these approaches presented minimal risk of genome insertion, the efficiency of iPSC generation is very low. Subsequently, iPSCs were generated using mRNAs encoding *POU5F1*, *SOX2*, *LIN28A* and *NANOG* [28], or mRNA encoding the Yamanaka factors [29]. Characterization of the iPSCs [29,30] generated by both mRNA-based methods showed higher identity of the global transcriptional signature with human ESCs by comparison to retroviral-derived iPSCs. Single nucleotide polymorphism analysis revealed that, in comparison to mRNA-derived iPSCs, those derived using the retroviral vectors had more mutations.

These observations suggested that an mRNA-based method is a safer choice for clinical applications than retrovirus-derived iPSCs. Although mRNA-based method generated transgene-free iPSCs with reasonable reprogramming efficiency (4%) [31], this protocol required daily transfection for 2 weeks. Recent efforts have simplified and optimized the mRNA-based reprogramming protocols [32,33]. However, once generated using mRNA technology, such iPSCs can be differentiated into clinical grade cardiomyocytes [34] using standard differentiation protocols. Furthermore, mRNA can be used to accelerate differentiation of iPSCs to the desired derivative. For example, mRNA encoding *ETV2* has been used to generate iPSC-derived ECs with high (90%) efficiency [35].

## 3. Employing mRNA to Directly Generate or Enhance Therapeutic Cells

It is also possible to use mRNA to directly generate therapeutic cells and/or enhance their proliferation, survival or functions. For example, mRNA encoding reprogramming or differentiation factors can be transfected into somatic cells that are derived from easily accessible somatic cells (e.g., skin fibroblasts) to directly generate cardiovascular cells ex vivo. Theoretically, these cells could be administered back to the patient by direct injection, or incorporated into biocompatible scaffolds. For example, fibroblasts have been transdifferentiated directly into cardiomyocytes in vitro and in vivo by overexpressing master regulators of cardiomyocyte lineage (i.e., *Gata4*, *Mef2c* and *Tbx5*) using a retroviral approach [36]. Similarly, we and others have used viral vectors overexpressing master regulators of endothelial lineage (e.g., *ETV2*, *FLI1*, *GATA2* and *KLF4*) to transdifferentiate fibroblasts into induced endothelial cells [37]. Rather than viral vectors, mRNA encoding master regulators of cardiovascular lineage may be used to achieve therapeutic transdifferentiation in a non-integrating manner that raises fewer safety concerns. Indeed, using mRNA to encode these endothelial transcription factors, we have successfully transdifferentiated human fibroblasts to endothelial cells with high transcriptional and functional fidelity to authentic human endothelial cells (Meng S and Cooke JP, unpublished data).

Although optimization of mRNA constructs and delivery vehicles are still necessary to introduce such therapies into the clinical practice, they hold great promise as proof-of-concept studies emerge. For instance, cardiac reprogramming of human mesenchymal stromal cells with an mRNA differentiation cocktail has been demonstrated recently [38]. Similarly, overexpression of human vascular endothelial growth factor A (*VEGFA*) with mRNA promotes endothelial specification of the human *ISL1*^+^ progenitors as well as their engraftment, proliferation and survival in vivo [39].

Mesenchymal stromal cells (MSCs) are available from different sources, such as the umbilical cord, bone marrow, liver, adipose tissue and multiple dental tissues [40]. These cells have the ability of self-renewal, differentiate into different cell lineages, migrate into the site of injury and secrete proteins which reduce inflammation and promote angiogenesis and tissue repair. Clinical trials are underway to assess the benefits of autologous MSCs in patients with ischemic syndromes or cardiomyopathy [41,42]. These trials are made more difficult by the heterogeneity of the quality of the biological product. A strategy to increase the therapeutic potency and homogeneity of the cell therapy would be through mRNA enhancement. Indeed, synthetic mRNA is being applied to engineer MSCs. Synthetic mRNA has been used to modulate the migratory properties of MSCs by temporal expression of homing proteins on the cell surface. In this way, mRNA-modified MSCs may be targeted to treat vascular inflammation. Specifically in one case [43], MSCs were transfected with three different synthetic mRNAs, *SELPLG*, *SLeX* and *IL10*, to enhance vascular targeting and the anti-inflammatory effect of MSCs, which were then systemically administered to the mice with LPS-induced vascular inflammation. *SELPLG* and *SLeX* are the ligands for P- and E-/L-selectin, respectively, and *IL10* acts as an immunosuppressive cytokine. The tethering capacity to the site of vascular inflammation was significantly improved in these mRNA-engineered MSCs. Furthermore, *IL10* levels were temporally increased in the inflamed region (the mouse ear) and were associated with superior local anti-inflammatory effect. In a similar approach [44], the therapeutic outcome of targeted delivery of MSCs triple-engineered with *SELPLG*/*SLeX*/*IL10* mRNAs was evaluated in a murine model of multiple sclerosis, a form of neurovascular inflammation. The engineered MSCs showed enhanced migration and adherence to inflamed brain microvascular endothelial cells and homing to the inflamed spinal cord. Additionally, *IL10* from these MSCs inhibited the proliferation of *CD4*+ T lymphocytes. In a model of brain ischemia, treatment of MSCs with mRNA encoding integrin a4 (*ITGA4*) facilitated their adhesion to endothelial *VCAM1* and improved the migration of MSCs into the ischemic region of the brain [45].

The C-X-C motif receptor 4 (*CXCR4*) is a chemokine receptor that binds to stromal derived factor-1 (*CXCL12*) expressed in inflammatory sites with high affinity. MSCs engineered with mRNA *CXCR4* showed improved cell migration toward *CXCL12* in transwell experiments, suggesting that transient initiation of chemotaxis can be triggered by mRNA-mediated chemokine receptor overexpression [46,47]. Thus, improvement of migration of MSCs by synthesized mRNA can enhance the capability for regeneration of damaged tissue.

## 4. Inflammatory Signaling in Nuclear Reprogramming and Transdifferentiation

The induction of pluripotency, as well as the transdifferentiation of one somatic cell to another, requires inflammatory signaling. For example, although the Yamanaka factors *OSKM* provide transcriptional direction, we now know that the retroviral vector also activates inflammatory signaling to increase chromatin accessibility, thereby permitting the Yamanaka factors to act on the promoter sequences of the network of genes required for pluripotency [48]. Whether one uses a viral vector or mRNA to induce pluripotency, activation of cell-autonomous inflammatory signaling is required for nuclear reprogramming to pluripotency.

The inflammatory signaling that is required for nuclear reprogramming is mediated by pattern recognition receptors (PRRs) that sense pathogen associated molecular patterns (PAMPs) or damage associated molecular patterns (DAMPs). Foreign mRNA activates toll-like receptors (TLRs) 3 and 7. Stimulation of these TLRs triggers inflammatory signaling pathways that activate NFkB, IRF-3 and IRF-7, which are known to induce genes encoding inflammatory cytokines and chemokines. In addition, these signaling pathways cause global changes in the expression of epigenetic modifiers that shift the balance between chromatin activators and repressors. For example, inflammatory signaling upregulates several members of the histone acetyltransferase (*HAT*) family, whereas those of the histone deacetylases (*HDAC*s) are downregulated [48].

In addition, this inflammatory signaling causes post-translational modifications of epigenetic modifiers that support the probability of an open chromatin state. The expression of inducible nitric oxide synthase (*NOS2*) is increased by *NFKB*, and it translocates to the nucleus, binding to and S-nitrosylating *RING1A* of the polycomb repressive complex 1 (PRC1) [49]. This S-nitrosylation of PRC1 causes it to disengage from the chromatin, removing this suppressive influence. Similarly, the *NuRD* complex is also S-nitrosylated by this inflammatory signaling process, preventing its de-acetylation and suppression of chromatin [50]. In addition to reactive nitrogen species, the transient generation of reactive oxygen species also appears to be required for efficient nuclear reprogramming [51].

Finally, a metabolic switch from oxidative phosphorylation to a glycolytic state is critical for transdifferentiation. Exogenous mRNA triggers a glycolytic switch, associated with citrate export from the mitochondria [52]. In the nucleus, there is an increase in citrate conversion to acetylcoA, thereby supplying the substrate for histone acetylation. Antagonism of this process abrogates the nuclear reprogramming required for a phenotypic switch.

Whereas most discussion of mRNA therapeutics emphasizes the need to reduce immunogenicity of the constructs, it is clear that some level of inflammatory signaling is required for exogenous mRNA to exert its effect when a change in cellular phenotype is desired. The effect of inflammatory signaling to facilitate an open chromatin configuration is critical for transcriptional activators to access their consensus sequences. On the other hand, excessive activation of inflammatory signaling may interfere with a desired phenotypic switch. Indeed, there appears to be a “Goldilocks zone” for optimal inflammatory signaling during nuclear reprogramming to pluripotency [50].

## 5. Employing mRNA to Reverse Cardiovascular Aging

One of the major determinants of cellular aging is telomere erosion. Telomeres of somatic cells become shorter with each division due to the “end-replication problem”, a process that is accelerated by oxidative stress. As cells approach their Hayflick limit, the telomere length reaches a critical threshold, triggers a DNA damage response and activates the *TP53*/*CDKN1A* pathway, with cell cycle arrest, senescence and degeneration [53,54,55]. Telomerase is a protein that reverses this process by extending telomeres. This protein is present in pluripotent stem cells, and to some extent adult stem cells, explaining the increased replicative capacity of these cells. Telomerase is generally not present in somatic cells, but can be reactivated in rapidly proliferating immune cells.

Our interest in using mRNA encoding human telomerase (*TERT*) as a therapeutic for vascular senescence arises from our work and others in the past 25 years demonstrating that vascular senescence is associated with an endotheliopathy that promotes atherosclerotic processes. For example, Chang and Harley measured telomere length from cadaveric human iliac and mammary arteries, finding telomere length shortens with increasing age [56]. Furthermore, at every age, telomeres were shorter in iliac arteries (which are more prone to atherosclerosis). We have shown that senescent human ECs generate less nitric oxide (NO), are more superoxide anion (O_2_^-^), synthesize more adhesion molecules, are more adhesive for monocytes and have reduced ability to proliferate and align with fluid shear stress [57]. These attributes promote vascular inflammation and atherogenesis. By contrast, when we overexpressed telomerase using a retroviral vector, telomere lengthening was associated with a reversal of the age-related endotheliopathy, and restoration of endothelial proliferative capacity and function.

**Figure 1:**
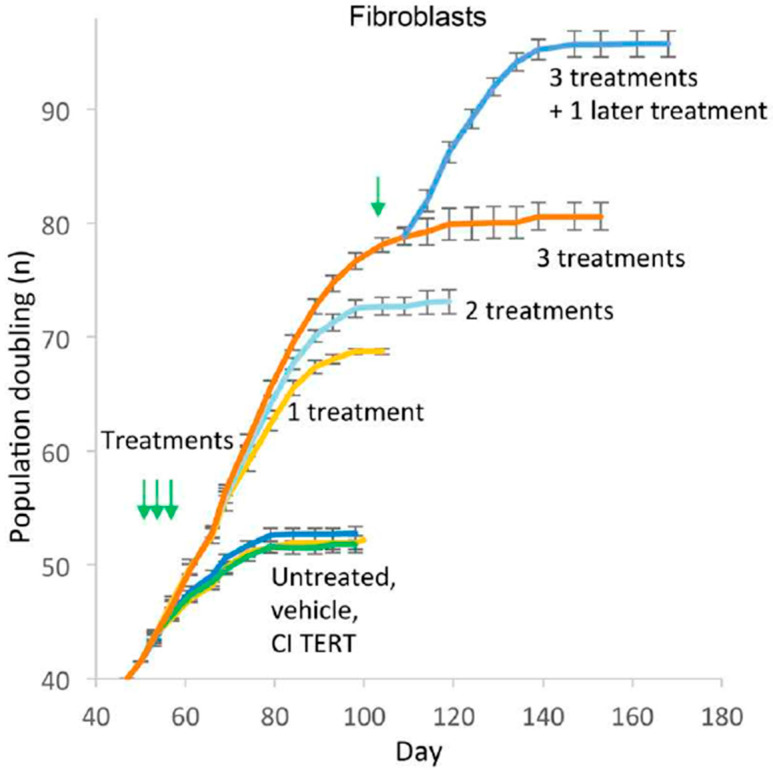
Growth curves of human fibroblasts treated once, twice or thrice in succession at 48 h interval with human telomerase (TERT) mRNA, catalytically inactive (CI) TERT mRNA, or vehicle only, shows increase in population doubling [58]. Green arrows indicate the time of treatment.

However, retroviral integration of telomerase in human cells raises the concern of unregulated growth. Accordingly, we used mRNA *TERT* to transiently express the telomerase, increase telomere length, enhance replicative capacity and reverse signs of senescence in human somatic cells [58]. After each transfection, the telomerase activity persists for no more than 72 h (by TRAP assay). Nevertheless, 1–3 treatments increase telomere length, population doublings and reduces the expression of the senescence marker, β galactosidase (β gal) in fibroblasts, endothelial cells and myoblasts, although the cells ultimately plateau in growth (Figure 1, [58]).

### Novel Therapeutic for Age-Related Diseases 

It was unexpected that transient transfection with *TERT* could have a long-term cellular benefit. Based on this work, we have developed a new mRNA therapeutic (codon-optimized, UTR-modified, HPLC-purified mRNA telomerase in lipid nanoparticles) to treat the endotheliopathy associated with vascular senescence. Our therapeutic has greater stability and less immunogenicity so as to increase transcription and reduce toxicity. Because endotheliopathy underlies many cardiovascular disorders, as well as other age-related diseases such as vascular dementia, peripheral arterial disease, nephrosclerosis, pulmonary fibrosis and impaired wound healing, correction of endotheliopathy due to senescence would be anticipated to mitigate or reverse many diseases and disorders associated with aging.

**Figure 2:**
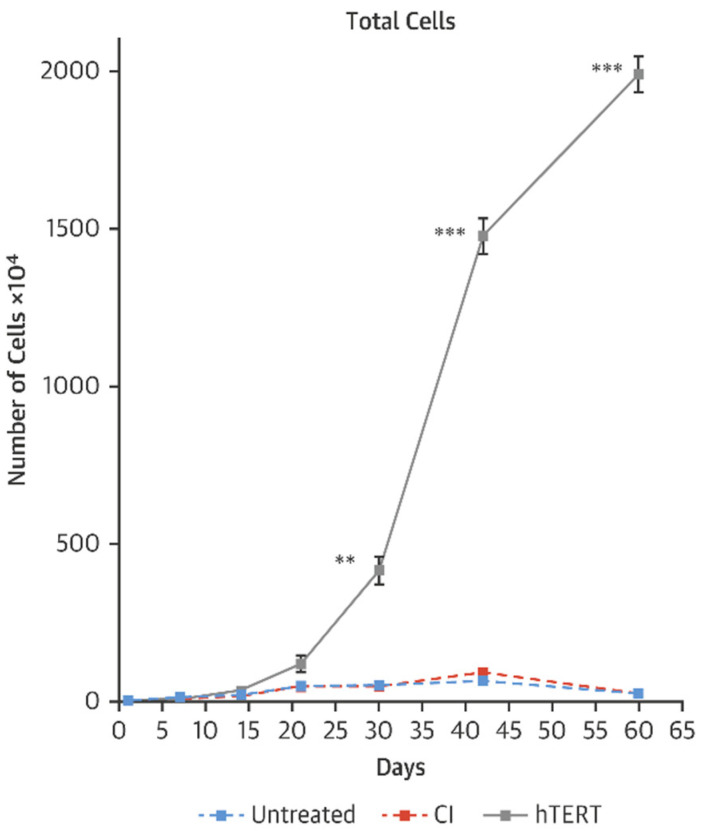
Growth curves of HGPS fibroblasts after 3 treatments with vehicle, transfected HGPS fibroblasts with human telomerase (TERT) or catalytically inactive (CI) TERT mRNA [59]. ** *p* < 0.01; *** *p* < 0.001.

As a model of accelerated aging, we have studied cells derived from children with Hutchison Gilford Progeria Syndrome (HGPS). We have observed that transient transfection using mRNA *TERT* of cells derived from HGPS children can increase telomere length, restore replicative capacity, reduce the expression of senescence markers and improve cellular functions in fibroblasts as well as iPSC-derived endothelial cells and vascular smooth muscle cells (Figure 2, [59]). Intriguingly, and not yet explained, mRNA *TERT* reduces progerin levels and improves nuclear morphology. Furthermore, we find that mRNA hTERT treatment of HGPS cells is superior to current therapy with the farnesyltransferase inhibitor lonafarnib as assessed by senescence markers, proliferation index and nuclear morphology [59,60].

One caveat for telomerase therapy is the possible risk for cancer. In about 85% of cancers, human telomerase is reactivated. However, it is very unlikely that transient expression of human telomerase using mRNA will increase the risk of cancer. We have abundant data that show that, after transient transfection with *TERT* mRNA, telomerase activity persists for less than 72 h [58]. Furthermore, although cells have improved replicative capacity, the growth curve of *TERT* treated cells has a normal pattern, i.e., there is a normal log phase of growth reaching a plateau, i.e., the treated cells are not immortalized. In fact, the treatment may reduce cancer risk: aged cells with short telomeres are more likely to undergo a “crisis”, which causes a DNA injury response, chromosome fusion and telomerase reactivation, leading to cancer [61,62,63]. This aberration might be prevented by telomere extension. Notably, our preliminary work suggests that mRNA telomerase treatment reverses the markers of DNA injury in HGPS, which would be expected to reduce the risk for oncogenesis.

## 6. Employing mRNA for Cardiovascular Regeneration

In addition to modifying therapeutic cells ex vivo, mRNA may be delivered directly into a tissue to have a therapeutic effect. This was first demonstrated in 1992, when Jirikowski and co-workers [10] injected synthetic mRNA encoding antidiuretic hormone (vasopressin) into hypothalamus of rats with a genetic deficiency of vasopressin. These animals have diabetes insipidus, which is characterized by difficulty concentrating urine, and the excretion of large volumes of diluted urine. Intrahypothalamic injection of vasopressin mRNA in these animals induced the synthesis of vasopressin protein and transiently reversed the disease. Since then, the feasibility of using mRNA to replace defective or missing proteins for therapeutic purposes was demonstrated in multiple studies and a variety of tissues [64,65,66,67,68]. Although the majority of the mRNA-based therapies are still in pre-clinical development, a growing number of candidates is reaching first-in-man trials [69,70,71].

The feasibility of direct intramyocardial injection was first reported in 2013 by Zangi et al. [72]. In this foundational work, *VEGFA* mRNA was injected into the ischemic region of murine myocardium at the time of coronary artery ligation. The local increase in *VEGFA* induced the expansion and directed differentiation of endogenous heart progenitors. Furthermore, this intervention markedly improved heart function and enhanced long-term survival of mice with experimental myocardial infarction (MI). Notably, *VEGFA* encoding plasmid DNA, unlike mRNA, significantly reduced survival of animals with MI in this study. The unexpected finding might be explained by the temporal differences in expression of *VEGFA* because prolonged exposure to *VEGFA* expressed from the plasmid DNA was associated with abnormal vascular permeability and myocardial edema.

The efficacy of *VEGFA* mRNA for heart regeneration after MI was subsequently confirmed in a large animal model by Carlsson at al [73]. In this study, MI was induced by a permanent ligation of the mid-left anterior descending coronary artery of mini pigs, and, 7 days after the initial surgery, naked mRNA was injected into the infarct and peri-infarct areas. Two months after injection, significant improvements were observed in left ventricular ejection fraction, contractility and myocardial compliance. Moreover, increased vessel density in the peri-infarct area and decreased myocardial fibrosis were noted in the hearts treated with *VEGFA* mRNA. Notably, the toxicity of the mRNA was also assessed in this study, and neither intradermal nor intravenous administration of the construct into both rats and cynomolgus monkeys increased serum levels of pro-inflammatory cytokines 24 h after injection.

These encouraging data led to the initiation of the first clinical trial of an mRNA therapeutic for cardiac regeneration, which was conducted by AstraZeneca (AZD8601) in collaboration with Moderna [74]. The EPICCURE study (NCT03370887) is a randomized, placebo-controlled, double-blind, multicenter, phase 2a clinical trial of the safety and efficacy of epicardial injections of *VEGFA* mRNA. The inclusion criteria specify patients with stable coronary artery disease and moderately decreased left ventricular ejection fraction who are undergoing coronary artery bypass grafting surgery [75]. The study is currently enrolling and is estimated to be completed in early 2023 [76]. Enrolled participants are to receive a placebo or either a low or high dose of AZD8601 (8 patients in each group) as 30 epicardial injections in a 10-min extension of cardioplegia. Injections will be targeted to ischemic but viable myocardial regions, which will be identified with positron emission tomography imaging. Improvement in myocardial blood flow will be an exploratory efficacy outcome, together with echocardiographic, clinical, functional and biomarker measures. The initiation of EPICCURE was preceded by a randomized, double-blind, placebo-controlled, phase 1 study in men with type 2 diabetes mellitus, where AZD8601 was given intradermally as a single ascending dose into the forearm skin with safety follow-up for 6 months [77]. The only causally treatment-related adverse event observed in the trial was an injection-site reaction of mild intensity, while local skin blood flow was significantly increased within 7 days after mRNA injection and correlated with amount of *VEGFA* protein concentration in cutaneous dialysate collected at the area. Similar findings were reported in the pre-clinical studies, where VEGF-A mRNA also facilitated healing of diabetic wounds [78,79].

Although *VEGFA* is currently the most advanced mRNA therapeutic candidate for cardiovascular regeneration, promising pre-clinical results have been reported for some other constructs targeting distinct molecular pathways. For instance, Chen at al. demonstrated that transcriptional co-activator yes-associated protein (*YYIAP1*) mRNA improved myocardial outcome after ischemia-reperfusion (IR) injury in mice [80]. *YYIAP1* mRNA significantly reduced the innate immune inflammatory response and cardiomyocyte survival in the injured myocardium, and 4 weeks later, heart function was improved and hypertrophic remodeling was suppressed.

Intramyocardial injection of synthetic mRNA encoding insulin-like growth factor-1 (*Igf1*) reduced apoptosis of cardiomyocytes after experimental MI in C57B1/6 mice [81]. The treatment augmented *Akt1* phosphorylation and decreased *Casp9* activity and TUNEL positive cells within the border zone 24 h post-MI. Notably, RNA uptake by the heart slice specimens cultured ex vivo was augmented in the presence of hypoxia compared to normoxic conditions. This may in part relate to cell membrane-enhanced polymer/RNA uptake following hypoxia.

The feasibility and benefits of manipulating IGF-1 signaling pathway with mRNA were also demonstrated by Zangi et al. [82]. However, their work revealed that whereas stimulation of *Igf1* receptor may enhance survival of cardiomyocytes and cardiac progenitors, it may also promote formation of epicardial adipose tissue in the injured heart. When mRNA encoding dominant-negative mutants of the *Igf1* receptor and insulin receptor substrate 1 (*Irs1*) were applied to the heart surface of adult mice as a gel to inhibit *Igf1* signaling in epicardial cells, it reduced the expression of adipogenic markers as well as the fraction of hearts with epicardial adipose tissue 28 days after MI.

Follistatin-like 1 (*FSTL1*) is increased in the ischemic myocardium. A modified form of *FSTL1* mRNA (with replacement of asparagine with glutamine in the N-glycosylation site at position 180) was sufficient and necessary to activate cardiomyocyte proliferation and limit cardiac remodeling post-MI, following a single injection of mRNA into the infarct border zone immediately after LAD ligation in mice [83].

Increased ceramide levels in mammalian heart during acute MI are associated with higher rates of myocyte death and impaired cardiac function [84,85,86]. Accordingly, mRNA encoding the enzyme acid ceramidase (*Asah1*) has been directly injected into the murine myocardium following MI induction. Such treatment was also associated with improved cardiac function, smaller scar size 28 days post-MI and longer survival [87].

Pyruvate kinase muscle isoenzyme 2 (*PKM*) is an isoenzyme of the glycolytic enzyme pyruvate kinase that is expressed in cardiomyocytes during development and immediately after birth, but not during adulthood [88]. Magadum et al. have recently discovered that *PKM* regulates the cardiomyocyte cell cycle and reduces oxidative stress damage through anabolic pathways and β-catenin [88]. In addition, these authors demonstrated that cardiomyocyte-specific *PKM* mRNA promoted cardiomyocyte cell division, enhanced cardiac function and improved long-term animal survival. To achieve specificity, kink-turn motif, a specific binding site for L7Ae protein [89,90], was added to 5′UTR of *PKM* mRNA. Subsequently, the modified *PKM* mRNA was co-delivered with mRNA encoding L7Ae which included cardiomyocyte-specific microRNA recognition elements (miR1-1 and miR-208a) within 3′UTR [91,92]. Because of these elements, translation of L7Ae was blocked specifically in cardiomyocytes, thereby allowing *PKM* expression (otherwise, L7Ae would interact with kink-turn motif on *PKM* mRNA and suppress its translation).

## 7. Future Perspectives

With improvements in mRNA constructs to improve their stability, enhance translation and promote delivery to the target tissue, the field of RNA Therapeutics is growing exponentially and advancing to clinical applications [93,94]. The recent success of mRNA vaccines against SARS-CoV-2 [3,4] has attracted great interest which will further accelerate the growth of this exciting frontier in medicine. RNA Therapeutics is a disruptive technology, as small biotech startups, as well as academic groups, can rapidly develop new and personalized constructs. Our group has long-standing expertise in designing and manufacturing RNA therapeutics for the scientific community and the demand for our services has increased substantially within the last five years. During these years, we learned that many small biotech companies and academic groups, who have innovative ideas for promising RNA therapeutics, lack key competencies to reach the clinic, such as manufacturing capabilities, delivery technologies or regulatory expertise. Our hospital-based RNA Therapeutics program operates in an environment tailored to accelerate novel therapeutics from conception to the clinic. Our fully integrated hospital-based RNA therapeutic program offers a single-entry point with consultation to ensure a seamless development and translation of RNA-based drugs into the clinic. The RNA core team helps investigators and small companies to develop new constructs and manufactures high quality research or clinical grade RNA. The RNA core is complemented by RNA biologists and bioinformaticians who provide fundamental expertise in RNA design. We are integrated with the Department of Nanomedicine, whose faculty have great expertise in the generation of novel nanoparticles for delivery of mRNA. Our facilities for clinical grade production of mRNA are overseen by an expert in cGMP manufacturing, who leads a team of cGMP-trained specialists and operators. Our GLP pre-clinical studies are led by our director of comparative medicine, who has extensive experience in designing and executing GLP preclinical studies. We have regulatory experts that provide assistance in planning regulatory roadmaps. For RNA therapeutics being introduced into the clinic, there is a seamless path from our first-in-man clinical trials unit to clinical trials in the nationally ranked Houston Methodist Hospital system. Our proprietary manufacturing processes have been licensed to a Contract Manufacturing Organization in the Houston area. This industry partner is capable of generating large batches of nucleic acid-based drugs for larger clinical trials and commercialization, thereby completing our assembly line to ensure a seamless transition from pre-clinical development and first-in-man studies to late-stage clinical trials and commercialization. To our knowledge, we are the only academic group with such an infrastructure, and we are excited to be working with small companies and academic research teams to facilitate the development of novel RNA therapeutics for cardiovascular regeneration and other applications.
